# Protective Effects of Total Saponins of *Aralia elata* (Miq.) on Endothelial Cell Injury Induced by TNF-α via Modulation of the PI3K/Akt and NF-κB Signalling Pathways

**DOI:** 10.3390/ijms20010036

**Published:** 2018-12-21

**Authors:** Ping Zhou, Weijie Xie, Yun Luo, Shan Lu, Ziru Dai, Ruiying Wang, Guibo Sun, Xiaobo Sun

**Affiliations:** 1 Beijing Key Laboratory of Innovative Drug Discovery of Traditional Chinese Medicine (Natural Medicine) and Translational Medicine, Institute of Medicinal Plant Development, Peking Union Medical College and Chinese Academy of Medical Sciences, Beijing 100193, China; zhoup0520@163.com (P.Z.); xwjginseng@126.com (W.X.); xlZhang2022@163.com (Y.L.); ginseng123@163.com (S.L.); athenadai219@163.com (Z.D.); shengjupan@163.com (R.W.); 2 Key Laboratory of Bioactive Substances and Resource Utilization of Chinese Herbal Medicine, Ministry of Education, Beijing 100193, China; 3 Key Laboratory of Efficacy Evaluation of Chinese Medicine against Glycolipid Metabolic Disorders, State Administration of Traditional Chinese Medicine, Beijing 100193, China; 4 Zhongguancun Open Laboratory of the Research and Development of Natural Medicine and Health Products, Beijing 100193, China

**Keywords:** atherosclerosis, tumour necrosis factor α, endothelial cell injury, inflammation, total saponins of *Aralia elata* (Miq.) Seem., apoptosis

## Abstract

Atherosclerosis is an arterial disease associated with inflammation. Hence, the discovery of novel therapeutic agents for suppressing inflammatory responses is urgent and vital for the treatment of atherosclerosis in cardiovascular diseases. The total saponins of *Aralia elata* (Miq.) Seem. (TAS) are the main components extracted from the Chinese traditional herb Longya *Aralia chinensis* L., a folk medicine used in Asian countries for treating numerous diseases, enhancing energy and boosting immunity. However, the protective effects of TAS against inflammation-triggered vascular endothelial dysfunction, a critical early event during the course of atherosclerosis, and the potential mechanisms of this protection have been not demonstrated. Accordingly, the aim of this study was to investigate the anti-inflammatory and anti-apoptotic effects and the protective mechanisms of TAS, and show how TAS ameliorates human umbilical vein endothelial cell (HUVEC) damage caused by tumour necrosis factor-α (TNF-α). The results indicated that TAS exerted cytoprotective effects by inhibiting TNF-α-triggered HUVEC apoptosis, mitochondrial membrane potential depolarisation, and the regulation of inflammatory factors (IL-6, MCP-1, and VCAM-1) while suppressing NF-κB transcription. Furthermore, this phenomenon was related to activation of the phosphoinositide 3-kinase (PI3K)/Akt signalling pathway. Blocking the Akt pathway with LY294002, a PI3K inhibitor, reversed the cytoprotective effect of TAS against TNF-α-induced endothelial cell death. Moreover, LY294002 partially abolished the effects of TAS on the upregulation of the Bcl-2 family of proteins and the downregulation of Bax protein expression. In conclusion, the results of our study suggest that TAS suppresses the inflammation and apoptosis of HUVECs induced by TNF-α and that PI3K/Akt signalling plays a key role in promoting cell survival and anti-inflammatory reactions during this process.

## 1. Introduction

Atherosclerosis is a multifactorial chronic inflammatory vascular disorder that occurs due to increased exposure to environmental and genetic risk factors. Increasing evidence indicates that the endothelial dysfunction and apoptosis caused by inflammation is a key inducer of atherosclerotic vascular disease [1]. Tumour necrosis factor (TNF-α), a critical inflammatory factor, has been shown to lead to the interaction of invading monocytes with vascular endothelial cells, which, in turn, causes endothelial apoptosis [2]. Human studies have revealed that TNF-α significantly induces extracellular matrix deposition on arterial walls, subsequently resulting in several intracellular signalling responses, such as the upregulation of interleukin (IL)-1β, IL-6, and matrix metallopeptidases [3,4]. In addition, inflammation causes iNOS and eNOS dysregulation, leading to endothelial dysfunction, which mainly manifests as an increase in adhesion molecules [5]. Activation of nuclear factor-κB (NF-κB) is indispensable for the stimulation of vascular TNF-α production and adhesion molecules, causing endothelial dysfunction under various pathological factors [6]. p65 forms heterogeneous dimers with members of the NF-κB family, and high levels of these dimers are found in the nucleus in endothelial cells, and plaques of patients with atherosclerosis [7].

*Aralia elata* (Miq.) Seem. is a well-known Chinese herbal medicine that is widely grown in north-eastern China (Heilongjiang, Liaoning and Jilin provinces), Far Eastern Russia, Japan and Korea. It exerts considerable regulatory effects, including antidiabetic, anti-tumour and anti-arthritic effects; it is also useful for non-alcoholic steatohepatitis and external bleeding due to injury [8,9]. The total saponins of *Aralia elata* (Miq.) Seem. (TAS), the main pharmacologically active ingredient extracted from AS stimulate heart activity, possess anti-myocardial ischaemic and anti-hypoxic activities, exhibit a strong anti-arrhythmic effect and exert protective effects against diabetic cardiomyopathy [8,9,10,11,12,13,14,15,16]. In addition, our group has studied *Aralia elata* (Miq.) Seem., including characterising TAS by using mass spectrometry (as shown in Supplementary Table S1 and Supplementary Figure S1), and has reported a positive inotropic effect of *Aralia elata* (Miq.) Seem. on canine myocardium and isolated rat cardiomyocytes, suggesting that *Aralia elata* (Miq.) Seem. can regulate Ca^2+^ in myocardial cells [9,10,13,14]. Previous studies by our group have also shown negative inflammatory responses and apoptotic activity of *Aralia elata* (Miq.) Seem. in liver tissue, and effects of TAS on septic cardiac dysfunction and its positive inotropic effects, in vitro and in vivo [13,17]. However, the effects and mechanism of TAS on endothelial dysfunction and apoptosis caused by inflammation have not been elaborated. 

The phosphoinositide 3-kinase (PI3K)–Akt signalling pathway is crucial in regulating cardiovascular function and injury [18]. Activating the PI3K/Akt pathway improves impaired vascular endothelial function [18,19]. A recent study has shown that TAS plays a protective role in lipopolysaccharide-induced acute cardiotoxicity, at least partially, through the PI3K/Akt signalling pathway [17]. We therefore hypothesise that TAS regulates inflammation through the PI3K/Akt signalling pathway, resulting in protection against atherosclerosis.

Considering the above evidence, the present study aimed to explore the effect of TAS and its molecular mechanisms on the effects of TNF-α-mediated vascular endothelial cell injury. In particular, this study focused on the intervention of TAS on the PI3K/Akt pathway. TAS plays an anti-inflammatory role, can directly reduce the transcription of NF-κB and reduce the production of relevant inflammatory factors, and it has a strong anti-apoptotic effect on endothelial cells after TNF-α treatment.

## 2. Results

### 2.1. TAS Reverses TNF-α-Induced Cell Viability Decrease and Caspase-3 Activation

In these experiments, HUVECs were pretreated with TAS for 2 h, followed by a 24 h co-incubation or co-treatment with various doses of TAS and TNF-α (50 ng/mL) in the drug administration groups; HUVECs were exposed only to TNF-α in the model groups. 

**Figure 1:**
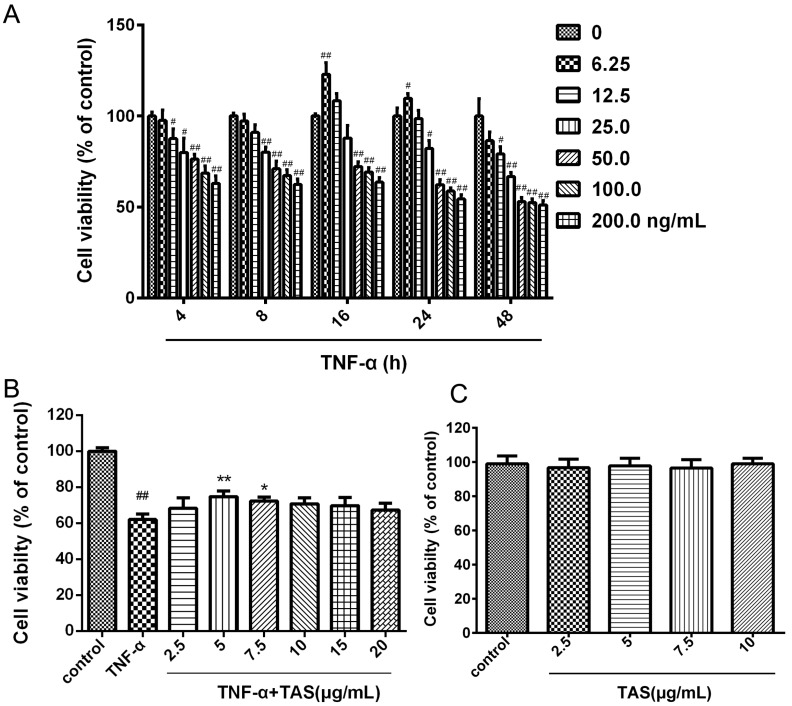
Total saponins of *Aralia elata* (Miq.) Seem. (TAS) protected human umbilical vein endothelial cells (HUVECs) against tumour necrosis factor (TNF)-α-induced injury. (**A**) HUVECs were incubated with TNF-α at different concentrations for various times (4, 8, 16, 24 and 48 h). Cell viability was determined using the MTT assay. (**B**) HUVECs were pretreated with TAS (2.5, 5, 7, 5, 10, 15 and 20 μg/mL) for 2 h, followed by treatment with TNF-α (50 ng/mL) for 24 h. Cell viability was measured using the MTT assay. (**C**) TAS administration alone showed no toxic effect. The data are presented as the mean ± SD of three independent tests. # *p* < 0.05, ## *p* < 0.01 versus the cont­rol group; * *p* < 0.05, ** *p* < 0.01 versus the TNF-α-incubation group.

The results showed that cell viability decreased in a time- and dose-dependent manner after TNF-α administration (Figure 1A). The MTT assay results showed that the viability of vascular endothelial cells subjected to TNF-α (50 ng/mL) decreased significantly compared with the control group, as previously reported [20]. When the cells were preincubated with TAS at various doses (0, 2.5, 5, 7.5, 10, 15, 20 μg/mL) for 2 h, followed by a 24 h incubation with TNF-α (50 ng/mL), cell viability changed markedly, suggesting that 5 and 7.5 μg/mL TAS demonstrated a significant cytoprotective effect, and that 5 μg/mL showed the strongest effect (Figure 1B). Then, we detected the cytotoxic effect of TAS; no significant differences between the TAS pretreatment and the control group were found (Figure 1C). Thus, 5 μg/mL TAS was used in subsequent experiments.

Moreover, 5 μg/mL TAS showed a significant cytoprotective effect against ox-LDL stimulus-induced endothelial cell injury (*p* < 0.01, Supplementary Figure S1); the cell viability increased markedly.

### 2.2. TAS Restores Mitochondrial Membrane Potential (ΔΨm)

**Figure 2:**
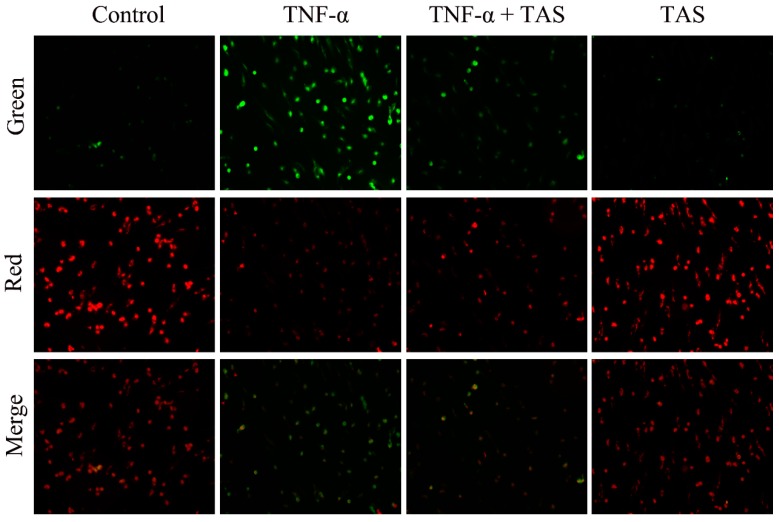
TAS restored mitochondrial transmembrane potential. HUVECs were preincubated with TAS (5 μg/mL) for 2 h, followed by treatment with TNF-α (50 ng/mL) for 24 h. Mitochondrial membrane potential was determined with JC-1 staining, which was observed using a fluorescence microscope. Image of fluorescence microscope on mitochondrial membrane potential (MMP) was obtained with 200 X of magnification.

TNF-α exerts a rapid toxic effect on mitochondria, which results in a decrease in ΔΨm and triggers respiratory chain electron transport process disorder, leading to the generation of apoptotic factors [21]. Consistent with the results of a previous work [20], the present work suggested that ΔΨm was decreased in TNF-α-treated cells, as shown by the green and red fluorescence intensity. As shown in Figure 2A,B, TAS substantially protected the mitochondrial membrane against the ΔΨm loss caused by 50 ng/mL TNF-α. The evident protective effects of TAS on the mitochondria indicated that the mitochondria are one of the target organelles of TNF-α in HUVECs.

### 2.3. TAS Ameliorates TNF-α-Induced Inflammation in HUVECs 

**Figure 3:**
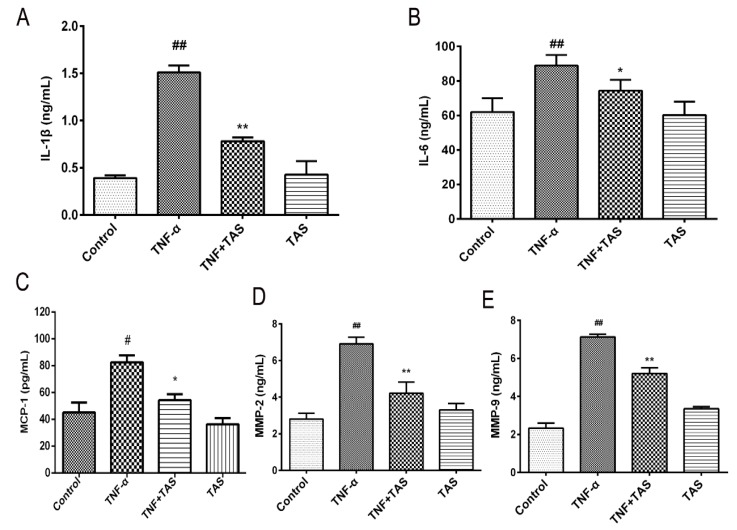
TAS suppressed the TNF-α-triggered inflammatory response in HUVECs. HUVECs were preincubated with TAS (5 μg/mL) for 2 h, followed by treatment with TNF-α (50 ng/mL) for 24 h. (**A**) IL-1β levels were measured by ELISA. (**B**) IL-6 levels were measured by ELISA. (**C**) MCP-1 expression after TAS treatment. (**D**) MMP-2 expression after TAS treatment. (**E**) MMP-9 expression after TAS treatment. The data are presented as the mean ± SD of three independent tests. # *p* < 0.05 and ## *p* < 0.01, versus the control group; * *p* < 0.05 and ** *p* < 0.01 versus the TNF-α-incubation group.

To verify the anti-inflammatory effect of TAS on HUVECs, cytokine secretion (including IL-1β, IL-6, MCP-1, MMP-2 and MMP-9) in response to treatment was measured. As shown in Figure 3A, B, C, D and E, the 50 ng/mL TNF-α-treatment group had a notable upregulated expression of IL-1β, IL-6, MCP-1, MMP-2 and MMP-9, compared with the vehicle group, which further suggested that TNF-α exhibits its cytotoxicity through the inflammatory process. Pretreatment with TAS effectively suppressed the production of these five indicators after TNF-α incubation. However, TAS administration alone had no effect on these inflammatory factors. 

### 2.4. TAS Alleviates TNF-α-Induced Endothelial Dysfunction

**Figure 4:**
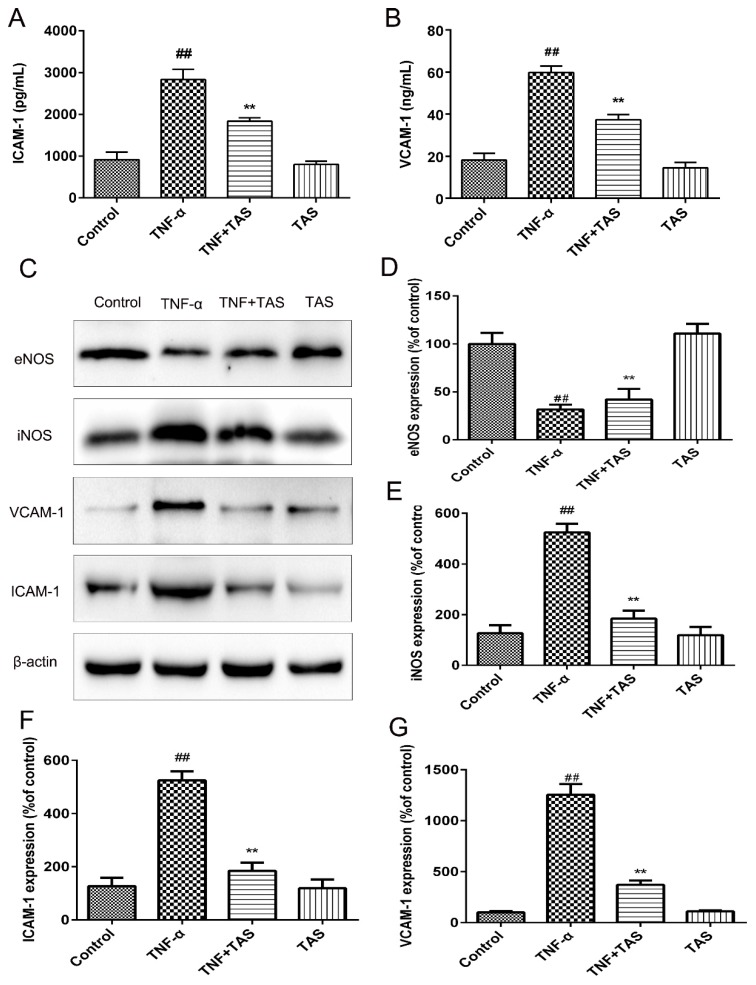
TAS reversed TNF-α-triggered endothelial dysfunction. HUVECs were preincubated with TAS (5 μg/mL) for 2 h, followed by treatment with TNF-α (50 ng/mL) for 24 h. (**A**) ICAM-1 expression was detected with ELISA. (**B**) VCAM-1 expression was detected with ELISA. (**C**) eNOS, iNOS, ICAM-1 and VCAM-1 expression were examined by Western blot analysis. (**D,E,F,G)** Quantitative analysis of ICAM-1, VCAM-1, eNOS and iNOS expression was performed. Each bar represents the mean ± SD of three independent experiments. ## *p* < 0.01 versus the control group; ** *p* < 0.01 versus the TNF-α-incubation group.

TNF-α pretreatment (50 ng/mL) induced significant enhancement of VCAM-1 and ICAM-1, and these effects were substantially suppressed by TAS preincubation (Figure 4A,B).

Western blot analysis results also suggested increases in VCAM-1 and ICAM-1 levels after TNF-α incubation that could be reversed by TAS treatment (Figure 4C,F,G). In addition, downregulation of iNOS expression and increased eNOS expression were observed after TAS preincubation compared with the TNF-α group (Figure 4C,E,G).

### 2.5. TAS Ameliorates TNF-α Induced Apoptosis in HUVECs

**Figure 5:**
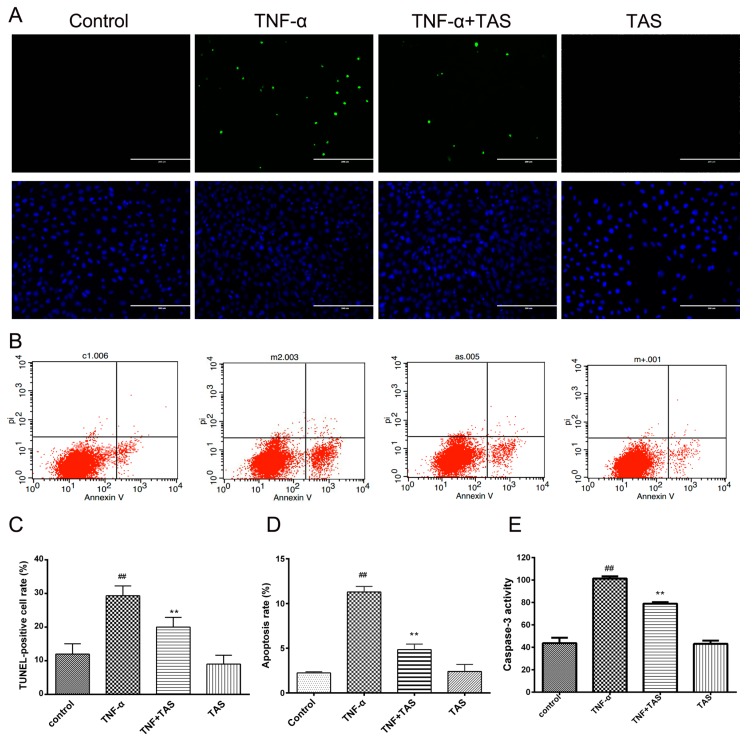
TAS suppressed TNF-α-induced apoptosis**.** HUVECs were preincubated with TAS (5 μg/mL) for 2 h, followed by treatment with TNF-α (50 ng/mL) for 24 h. HUVEC apoptosis was assessed by (**A**) TUNEL staining and (**B**) annexin V/propidium iodide (PI) staining. (**C**) Quantitative analysis of the ratio of TUNEL-positive cells. (**D**) Quantitative analysis of the ratio of apoptotic cells. (**E**) Cells were preincubated with TAS (5 μg/mL) for 2 h, followed by treatment with TNF-α for 24 h. Caspase-3 activity was examined with a fluorescent labelling kit using a microplate reader. The data are expressed as the mean ± SD of three independent experiments. ## *p* < 0.01 versus the control group; ** *p* < 0.01 versus the TNF-α-incubation group. Scale bars, 100 μm.

Annexin V and propidium iodide (PI) double staining was used to detect early apoptotic cells, which is an accepted method [22]. The apoptotic rate of HUVECs was examined by flow cytometry. The ratio of early-stage apoptotic cells of the TNF-α-treated group was enhanced dramatically. By contrast, TAS administration blocked the phenomenon. TAS treatment alone had no effect on apoptotic cells (Figure 5B,D). The above conditions confirm that TAS could protect endothelial cells from TNF-α-caused apoptosis.

Moreover, TUNEL staining displayed an increased rate of DNA fragmentation in HUVECs caused by TNF-α incubation, and TAS treatment significantly reversed this phenomenon (Figure 5A,C). These results verified that TAS could protect HUVECs from TNF-α-triggered apoptosis.

Activated caspase-3 plays a critical role in apoptosis [23], and Figure 5E shows that TNF-α substantially activated caspase-3 in cells compared with the vehicle group. TAS administration significantly decreased cleaved caspase-3 levels, highlighting the crucial effect of activated caspase-3 on TNF-α-induced cell death and the potential anti-apoptotic effect of TAS. There was no significant difference between TAS treatment alone and the control group.

### 2.6. TAS Regulates the Expression of Apoptosis-Related Proteins 

**Figure 6:**
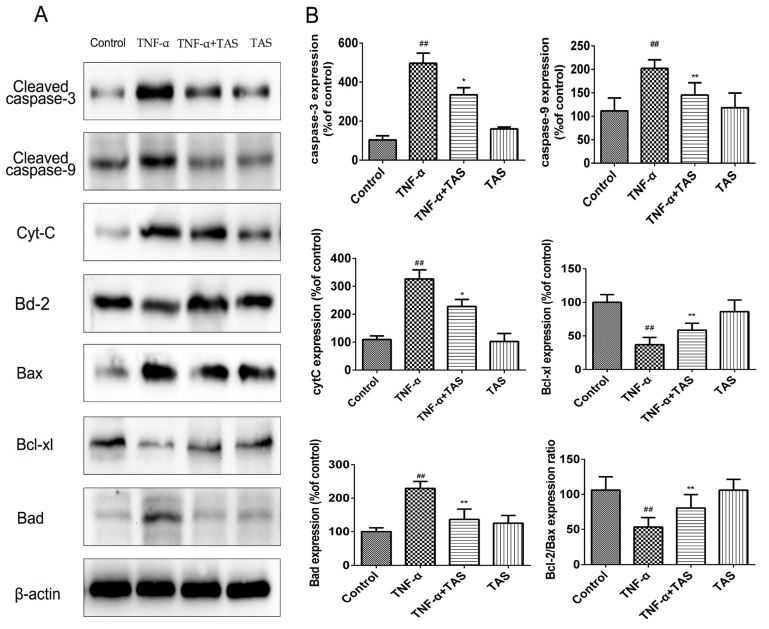
TAS modulated apoptosis-related proteins in HUVECs. HUVECs were preincubated with TAS (5 μg/mL) for 2 h, followed by treatment with TNF-α (50 ng/mL) for 24 h. (**A**) The protein expression of the Bcl-2 family and caspase protein family. (**B**) Quantitative analysis of the corresponding protein expression. The data are expressed as the mean ± SD of three independent experiments. ## *p* < 0.01 versus the control group; * *p* < 0.05 and ** *p* < 0.01 versus the TNF-α-treatment group.

To clarify the potential mechanisms by which TAS exerted its protective effect on apoptosis after TNF-α exposure, Western blot analysis was performed using HUVECs. The enhancement of caspase and related cytokines is essential for apoptosis in various physiological and pathological activities. In accordance with the results of fluorescein-based active caspase-3 staining, the results of Western blot showed an upregulation of caspase-3 by TNF-α treatment; in addition, caspase-9 and cytochrome C expression levels were enhanced, and this effect was reversed by TAS treatment (Figure 6). Furthermore, the expression level of caspase-8 was also determined by Western blotting, and it showed TAS treatment weaken the upregulation of caspase-8 induced by TNF-α treatment (Supplementary Figure S2).

The Bcl-2 protein family, including Bax, Bcl-2, Bad, and Bcl-xl, plays a pivotal role in the regulation of endotheliocyte apoptosis [24]. Our results suggested that the expression levels of the cytoprotective proteins Bcl-2/Bax and Bcl-xl were decreased after TNF-α incubation; this effect was reversed by pretreatment with TAS (Figure 6), while Bad exhibited the opposite expression pattern. In conclusion, our study showed that TAS can enhance the level of anti-apoptotic proteins and decrease the expression of pro-apoptotic proteins to prevent endothelial cell apoptosis. 

### 2.7. TAS Regulates the NF-κB Signalling Pathway 

**Figure 7:**
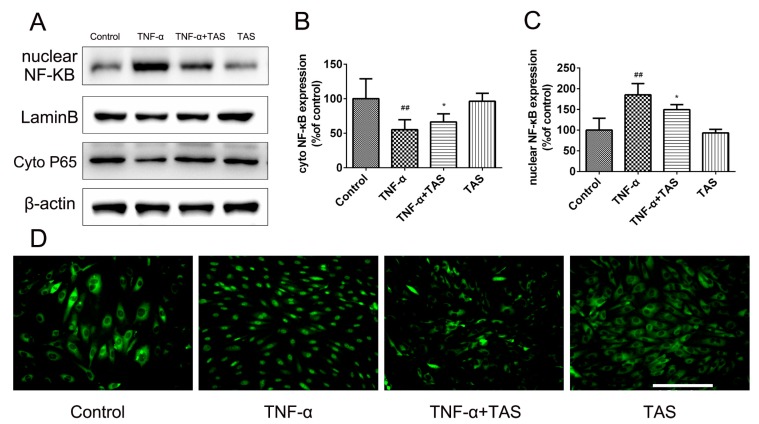
TAS protected against HUVEC damage triggered by TNF-α via the NF-κB pathway. HUVECs were preincubated with TAS (5 μg/mL) for 2 h, followed by treatment with TNF-α (50 ng/mL) for 24 h. (**A**). The levels of NF-κB p65 in the nucleus and cytoplasm. (**B**) Quantitative analysis of NF-κB p65 expression in the cytoplasm. (**C**) Quantitative analysis of NF-κB p65 expression in the nucleus. (**D**) The location of NF-κB p65 was observed by immunofluorescent staining. The data are expressed as the mean ± SD of three independent experiments. ## *p* < 0.01 versus the control group; * *p* < 0.05 versus the TNF-α-treatment group. Scale bars, 50 μm.

NF-κB plays a crucial role in the reaction of inflammation, resulting in the transcription of genes involved in endothelial injury and inflammation [25,26]. The NF-κB signalling pathway in vascular endothelial cells is activated by TNF-α stimulation [27]. In our study, TNF-α treatment induced a significant decrease in IκB-α expression. In addition, NF-κB p65 showed a decreased expression in the cytoplasm, but an increased expression in the nucleus after TNF-α-preincubation. However, TAS significantly reduced the translocation of p65 from the cytoplasm to the nucleus (Figure 7A–C). The immunofluorescence results confirmed this phenomenon, indicating that the anti-inflammatory effect of TAS occurs via the NF-κB pathway (Figure 7D).

### 2.8. TAS Exert Protective Effects via Activation of the PI3K/Akt Pathway

**Figure 8:**
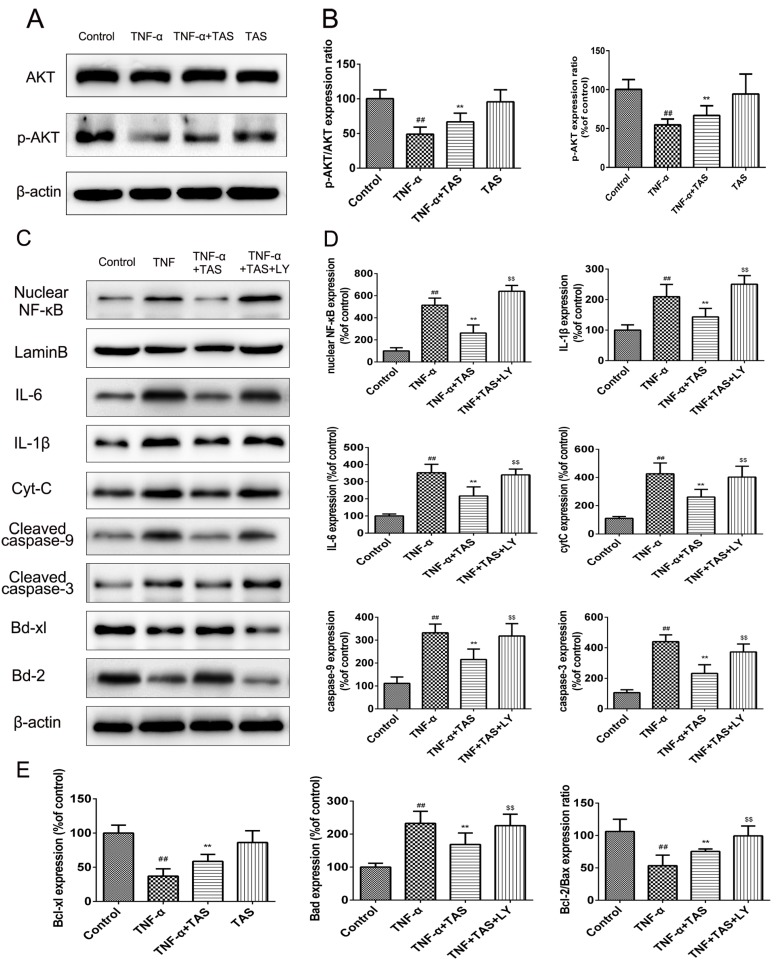
TAS exerted a protective effect via activation of the PI3K/Akt pathway. HUVECs were preincubated with TAS (5 μg/mL) for 2 h, followed by treatment with TNF-α (50 ng/mL) for 24 h. (**A**) Akt expression and its phosphorylation were measured by Western blot. (**B**) Quantitative analysis of Akt and p-Akt expression. (**C**) Effect of LY294002 (PI3K inhibitor) on the expression of related proteins in HUVECs after TAS treatment. (**D** and **E**) Quantitative analysis of corresponding protein expression levels. The data are expressed as the mean ± SD of three independent experiments. ## *p* < 0.01 versus the control group; ** *p* < 0.01 versus the TNF-α-treatment group; $ *p* < 0.05 and $$ *p* < 0.01 versus the TNF-α and TAS groups.

PI3K/Akt signalling plays a critical role in modulating the NF-κB signalling pathway and TNF-α-induced apoptosis in various cells [28,29]. To clarify the underlying signalling pathways involved in the anti-inflammatory and anti-apoptotic effects of TAS, Western blot analysis was conducted to detect Akt phosphorylation. The results (Figure 8A,B) showed that TAS increased Akt phosphorylation that was inhibited by TNF-α, which indicated the predominant function of Akt signalling in this process.

Afterwards, we detected whether the addition of LY294002, a PI3K inhibitor, could attenuate the effect on the NF-κB signalling pathway and apoptosis-associated proteins. Western blot analysis results showed that HUVECs treated with TNF-α and TAS exhibited enhanced expression of Bcl-2 and Bcl-xl, which showed no significant difference in the TNF-α treatment-alone group. Nevertheless, the protein expression levels of these two proteins were suppressed after PI3K/Akt inhibition in HUVECs incubated with LY294002. The levels of apoptosis-related proteins, including Bax, caspase-3 and caspase-9, were further investigated. TAS inhibited the TNF-α-induced upregulation of the abovementioned molecules, which was enhanced substantially in the presence of LY294002 (Figure 8C–E). Additionally, TAS pretreatment further partially inhibited nuclear NF-κB expression and that of related inflammatory factors, which were elevated by LY294002. In summary, these results demonstrate that TAS inhibited TNF-α-induced apoptosis and the inflammatory reaction in a PI3K/Akt-dependent manner.

## 3. Discussion

Endothelial cell damage is considered a crucial step for the pathogenesis of atherosclerotic vascular disease including plaque formation [30]. However, in the last 20 years, a growing amount of evidence has demonstrated that inflammatory processes play a major role from the very beginning to the ultimate complication of atherothrombosis [31]. Moreover, they may cause plaque rupture by production of pro-inflammatory cytokines, such as IL-6, IL-1, tumour necrosis factor-α (TNF-α) and ox-LDL. Hence, there is a growing interest towards anti-inflammatory agents as preventive or curative treatments of atherothrombosis [31,32]. 

TAS, the main component of the herbal extracts from the Longya *Aralia chinensis* L., exhibits a series of pharmacological functions, including anti-inflammatory, antioxidant, hypolipidaemic and antidiabetic properties [8]. In our research, we first elucidated the protective effects of TAS against TNF-α-triggered vascular endothelial cell damage and demonstrated that TAS prevented endothelial dysfunction through exerting anti-inflammatory and anti-apoptotic action. 

Conforming to previous results [20], our present study confirmed that TNF-α treatment induced notable endothelial cell death. However, surprisingly, we suggested in the current study that TAS administration for 2 h prior to TNF-α incubation dramatically prevented the HUVECs from TNF-α-triggered apoptosis by suppressing the overexpression of inflammatory factors, decreasing the viable apoptotic cell ratio, suppressing DNA fragmentation, and reducing caspase-3 activation. In addition, inflammatory cytokine-induced injury is a critical aetiology of TNF-α-induced endothelial damage [33]. Our results verified that TNF-α administration substantially increased the levels of inflammatory cytokines, such as interleukins, MCP-1, MMP-2, and MMP-9, that mainly contribute to the development of vessel dysfunction in atherosclerosis. Interestingly, our data also indicated that pretreatment with TAS reversed this damage due to inflammatory processes. For example, the increased levels of vascular adhesion factors, including VCAM-1 and ICAM-1, were notably inhibited by TAS. The rescue effect of TAS treatment on the TNF-α-induced decrease in cell viability was not pronounced, but its protective effects on apoptosis and inflammation were more significant, most likely because the endothelium regulates the vascular system with roles in processes, such as haemostasis, cell cholesterol, hormone trafficking, signal transduction and inflammation [34]. Furthermore, TAS treatment could reduce caspase-3 and caspase-9 activation; the expression level of caspase-8 was also determined by Western blotting, and it showed TAS treatment weakened the upregulation of caspase-8 induced by TNF-α treatment (Supplementary Figure S2). However, it needs to be further explored how TAS treatment regulates the caspase-8 activation. 

Pro-inflammatory factors play critical roles during the course of immunisation and inhibit cardiac contractile function via improving NO levels [35]. Accumulating studies have suggested that NO directly affects vascular tension and mitochondrial dyspnoea, followed by mediating the release of inflammatory factors [36]. TNF-α and pro-inflammatory cytokines consequently promote iNOS activity in endotheliocytes [37,38]. The iNOS level increase in the process of cardiac function impairment, as well as vascular inflammation, and further increase the expression of NO, which mainly contributes to cardiac failure. In the current research, we found that TNF-α incubation led to disorders of eNOS and iNOS in the endothelium system, which could be notably reversed after TAS administration. Additionally, TAS showed a significant cytoprotective effect against ox-LDL stimulus-induced endothelial cell injury (Supplementary Figure S1). All of these indicated that TAS exerted significant cytoprotective effects against endothelial cell damage via inhibiting inflammation.

NF-κB activation is essential for the regulation of key cytokines involved in the inflammatory processes that play critical roles in atherogenesis [39]. One of the highlights of this study is the discovery that TAS inhibited the nuclear translocation of NF-κB. Fluorescence microscopy and Western analysis indicated the downregulation of NF-κB p65 in the cytoplasm and its upregulation in the nucleus after TNF-α incubation; however, TAS administration reversed this phenomenon. The above effects clarified that TAS significantly suppresses TNF-α-triggered NF-κB transcription, the principle event contributing to the inflammatory reaction.

Accumulating evidence has shown that activation of the Akt-associated signalling pathway is related to endothelial cell function [40,41]. Meanwhile, the activation of PI3K and its downstream Akt molecules was reported to inhibit endotheliocyte apoptosis and promote cell survival by improving endothelial homeostasis and impaired endothelium [40]. A recent study demonstrated that TAS exerts a protective effect against non-alcoholic fatty liver disease in a manner associated with Akt activation [42]. In the current study, we also found that the protective mechanism of TAS correlates with Akt phosphorylation; this hypothesis was supported by the addition of LY294002, which inhibits PI3K and dramatically reversed TAS-triggered Akt phosphorylation and blocked the protective effect of TAS during TNF-α-triggered endothelial damage (Figure 8). These data demonstrated that the positive effect of TAS on TNF-α-mediated HUVEC death was at least partly regulated via PI3K/Akt signalling.

The Akt-related signalling pathway is indispensable for cellular activity because it regulates the promotion and downregulation of mitochondria-mediated apoptosis proteins, such as the Bcl-2 family members [43]. The Bcl-2 family is a major contributor to a large family of apoptotic proteins, including proteins that promote apoptosis, such as Bad, Bak and Bax, and proteins that inhibit apoptosis, such as Bcl-xl and Bcl-2 [44]. Cell fate is determined by the modulation of these two types of Bcl-2 family members [45]. To explore whether the promotion of PI3K/Akt signalling after TAS treatment helps to maintain cell viability through regulating the level of Bcl-2 family proteins, the levels of apoptosis-blocking members and the apoptosis-inducing element Bax were determined. The current study suggests that TNF-α causes a decrease in Bcl-2 and Bcl-xl protein levels and an increase in the Bax protein level. Furthermore, after TAS administration, the expression of Bcl-2 and Bcl-xl was significantly upregulated, while Bax expression was dramatically reduced. Interestingly, blocking PI3K/Akt signalling using a specific inhibitor reversed the above phenomena. In sum, PI3K exerts a protective effect by restoring the imbalance between anti-apoptotic proteins and the pro-apoptotic protein upon TAS administration after TNF-α-mediated HUVEC injury.

**Figure 9:**
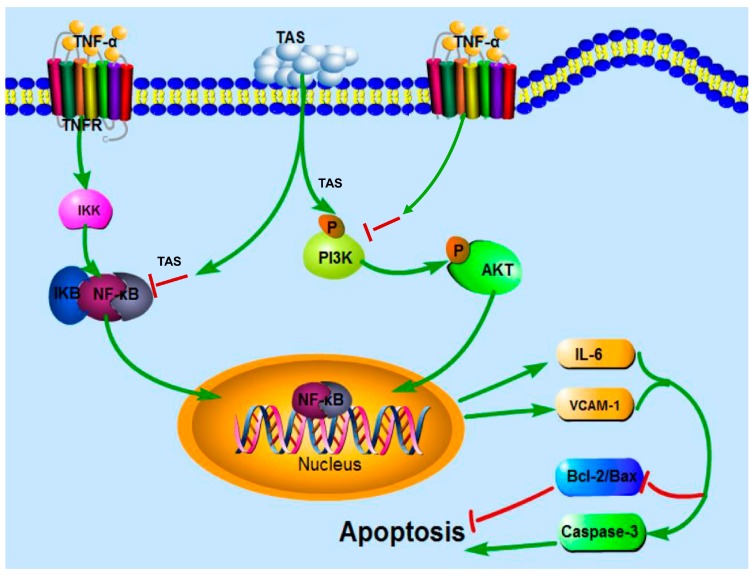
Schematic diagram of the mechanism by which TAS protects against TNF-α-mediated HUVEC injury. TAS suppresses the inflammation and apoptosis of HUVECs induced by TNF-α via PI3K/Akt signalling and NF-κB signalling pathways. TAS stands for total saponins of *Aralia elata* (Miq.).

In conclusion, as shown in Figure 9, our results first showed that pretreatment with TAS dramatically alleviated TNF-α-induced endothelial inflammatory factors release, disorders between iNOS and eNOS, and NF-κB translocation. TAS attenuated endothelial dysfunction through the activation of PI3K/Akt signalling, followed by regulating the levels of inflammatory factors and the expression of Bcl-2 family proteins, such as Bax and Bcl-2, which led to decreased levels of activated caspase-3. The rescue effect of TAS treatment on the TNF-α-induced decrease in cell viability was not pronounced, but its protective effects on apoptosis and inflammation were more significant, most likely because cell viability is affected by more factors. Moreover, mounting evidence links atherosclerosis to endothelial dysfunction; in fact, the endothelium regulates the vascular system with roles in processes such as haemostasis, hormone trafficking, signal transduction and inflammation [34]. The above findings demonstrate the potential of TAS to treat endothelial injury. Nevertheless, further studies are needed to investigate the mechanisms of action.

## 4. Materials and Methods

### 4.1. Materials 

TAS roots were obtained and authenticated from the Academy of Chinese Medical Sciences (Changchun, China). TAS extraction, separation and quality control are consistent with previous reports [13]. Human recombinant TNF-α was obtained from Sigma Aldrich (St. Louis, MO, USA). Endothelial cell culture medium (VascuLife) was purchased from LifeLine Cell Technology (Frederick, MD, USA). 3-(4,5-dimethylthiazol-2-yl)-2,5-diphenyltetrazolium bromide (MTT) and fluorescent dye JC-1 were acquired from Enzo Life Sciences (Farmingdale, NY, USA). An annexin V/propidium iodide (PI) apoptosis detection kit and the terminal deoxynucleotidyl transferase biotin-dUTP nick end labelling (TUNEL) assay kit was acquired from Roche Diagnostics GmbH (Mannheim, Germany). ELISA kits for determining ICAM-1, VCAM-1, IL-1β, IL-6, matrix metallopeptidase-2 (MMP-2) and matrix metallopeptidase-9 (MMP-9) levels were acquired from Expandbio (Beijing, China). Primary and secondary antibodies and protein extraction kits were purchased from Abcam (Cambridge, MA, USA). The Western blot detection assay kits were obtained from Pierce Biotechnology (IL, USA). Antibodies against p-Akt (ab131443), Akt (ab106693), BCL-2 (ab59348), BAX (ab32503), cleaved-caspase-3 (ab4051), cleaved-caspase-9 (ab52298), cytoC (ab13575), ICAM-1 (ab20), Bad (ab32445), Bcl-xl (ab32370), iNOS (ab3523) and eNOS (ab76198) were obtained from Abcam, while the VCAM-1 antibody (#13662) was purchased from Cell Signaling Technology (Danvers, MA, USA). Other reagents were obtained from Sigma Aldrich (St. Louis, MO, USA). The PI3K inhibitor LY294002 was acquired from Calbiochem (San Diego, CA, USA). All chemicals were at least analytical grade.

### 4.2. Cell Culture and Treatment

The umbilical cords of new-borns were donated by the Haidian District Maternal and Child Health Hospital, Beijing, China, and the research protocol was approved by the Ethics Committee of Peking Union Medical College (Beijing, China) on 18 December 2015 (SLXD-20151214). According to the national standard of the People’s Republic of China (GB14925-2010), this study was conducted in compliance with the Declaration of Helsinki. The isolation methods of human umbilical vein endothelial cells (HUVECs) were the same as those described previously [19]. Then, 2 to 4 passages of cells were used for this study. The TAS mother liquor was prepared in DMSO and then diluted with serum-free medium as required before use. TNF-α was prepared to the desired concentrations immediately before treatment using serum-free medium. In these experiments, HUVECs were pretreated with TAS for 2 h, followed by a 24 h co-incubation or co-treatment with various doses of TAS and TNF-α (50 ng/mL) in the drug administration groups; in the model groups, HUVECs were exposed only to TNF-α. 

### 4.3. Cell Activity Analysis

HUVECs viability was evaluated using MTT chemosensitivity testing with a microplate reader. Cells were plated in 96-well gelatine-coated plates in a volume of 100 μL per well (cell density, 1 × 10^5^ cells/mL), and the cells were allowed to adhere and grow for 24 h. HUVECs were pretreated with TAS, washed with PBS, and then exposed to TNF-α. Afterwards, MTT (1 mg/mL) was supplemented to each group, followed by incubation for 4 h at 37 °C. A total of 100 μL of DMSO was added to dissolve the formazan crystals. Cell viability was reflected by absorbance, which was measured at 570 nm using a microplate reader (SpectraFluor, Tecan, Sunrise, Austria) after 2 min of shaking. Cell viability was expressed as a percentage of the control value. Each experiment was performed in quintuplicate using three independent cultures.

### 4.4. Quantification of Apoptosis

Annexin V/PI assay kits (Invitrogen, CA, USA) were used to detect the proportions of viable and apoptotic cells in different treatment groups. HUVECs (1 × 10^5^ cells/mL) were plated in collagen-coated six-well plates. After incubation with TAS (5 μg/mL) for 2 h, the cells were washed with PBS and treated with TNF-α (50 ng/mL) for 24 h. The cells were harvested, washed with cold PBS, and then conditioned with 1× annexin V working solution, which contains PI (1 μg/mL final concentration), in the dark, at room temperature, for 15 min. Four hundred microliters of 1× binding buffer was added, and the cells were then analysed using a FACSCalibur flow cytometer (BD Biosciences, CA, USA). The results are expressed and analysed from three independent experiments.

### 4.5. TUNEL Assay

DNA fragmentation and cell apoptosis were examined with a TUNEL staining kit according to the manufacturer's protocol. Briefly, after all processes, endothelial cells were washed with PBS and fixed in 4% buffered formaldehyde for 30 min, and then incubated with a methanol solution with 0.3% H_2_O_2_. After rinsing with PBS, the cells were incubated with a permeabilising solution containing 0.1% Triton X-100 for 10 min. Then, the TUNEL reaction mixture was added to each well, followed by incubation for 1 h at 37 °C in the dark. Thereafter, the samples were washed with PBS and counterstained with diamidino-2-phenylindole (DAPI). After washing with equilibration buffer, images were captured using a fluorescence microscope (Leica DM4000, Frankfurt, Germany). The TUNEL-positive cell ratio was calculated according to previous methods [46]. The statistical results are expressed from three independent experiments.

### 4.6. Measurement of Mitochondrial Transmembrane Potential ΔΨm

JC-1 (Enzo Life Sciences International, USA) staining was conducted to evaluate the changes in mitochondrial membrane potential. HUVECs (1 × 10^5^ cells/mL) in each group cultured in 12-well cell culture plates were preincubated with 5 μg/mL TAS for 2 h, followed by 50 ng/mL TNF-α treatment. The cells were collected, washed, and then incubated with JC-1 dye working fluid in the dark for 30 min at 37 °C. After rinsing twice with PBS, images of the stained cells were captured using a fluorescence microscope (Leica Q9, Frankfurt, Germany). Representative images from at least three independent experiments are shown.

### 4.7. ELISA

The levels of iNOS, eNOS, and a series of cytokines were assessed using ELISA kits (Nanjing Jiancheng Bioengineering Institute, Nanjing, China) according to the manufacturer’s instructions. For the detection of the abovementioned factors, the supernatants of the cells were harvested after treatment with the appropriate drugs and vehicle, and then determined according to the protocols provided by the manufacturer. Three independent experiments were performed. 

### 4.8. Immunofluorescence

To detect the subcellular localisation of NF-κB by immunofluorescence, HUVECs (1 × 10^5^ cells/mL) were cultured in a 24-well plate for 24 h, followed by pretreatment with TAS or TNF-α. After fixation in 4% paraformaldehyde for 10 min, 0.1% sodium citrate and Triton X-100 solution were used to permeabilise cells. Afterwards, 1% BSA was added for 1 h, and then the samples were incubated with the NF-κB primary antibody for 12 h at 4 °C. After rinsing with PBS, corresponding secondary antibodies conjugated with fluorescein isothiocyanate (FITC) fluorochromes were added to each well at room temperature. After a 30 min incubation at room temperature, images of stained cells were obtained using a fluorescence microscope. Representative images that were analysed from at least three independent experiments are shown.

### 4.9. Detection of Caspase-3 Activity

A fluorescence staining kit (BioVision, CA, USA) was used to detect the activation degree of caspase-3. Briefly, after all processes, 50 μL of precooled buffer was added to each group for 10 min; then, 50 μL of 2× reaction buffer (containing 10 mM dithiothreitol) and 5 μL of DEVD-7-amino-4-trifluoromethylcoumarin were added to each well, followed by incubation at 37 °C for 2 h. Under the conditions of 400 nm excitation wavelength and 505 nm emission wavelength, the fluorescence intensity was detected. Three independent experiments were performed independently.

### 4.10. Preparation of Cytosolic and Nuclear Proteins

Nuclear and cytosolic proteins were separated using a nuclear and cytoplasmic extraction kit (CWBIO, Beijing, China). HUVECs were briefly washed with cold PBS, followed by dissociation in cold buffer A containing 1% phosphatase inhibitor and protease inhibitor cocktail. The supernatant containing cytosolic proteins was collected after centrifugation at 12,000 rpm for 15 min at a low temperature. Then, the nucleoprotein lysate containing protease inhibitor cocktail was added to the remaining sediment for further cleavage, and the samples were placed on ice for half an hour. Subsequently, the supernatant containing nuclear proteins was transferred to the corresponding test tube after centrifugation at 12,000 rpm for 15 min. The BCA assay kit was used to detect protein concentration according to the provided procedure. Then, the loading buffer was added according to a quarter of the amount of protein solution, followed by boiling for 5 min. The cytosolic and nuclear proteins were preserved at −80 °C until use.

### 4.11. Western Blot Analysis 

HUVECs were preincubated with TAS (5 μg/mL for 2 h) or LY294002 (50 μmol/L for 2 h), followed by a 24 h exposure to TNF-α (50 ng/mL). Total cellular proteins and the nuclear proteins were extracted using the nuclear and cytoplasmic protein extraction kits as mentioned above, followed by protein concentration determination using a BCA kit. Equivalent concentrations of protein samples from different groups were separated by electrophoresis and transferred to a membrane. Next, the membranes were blocked for more than 2 h in non-fat milk powder solution at approximately 25 °C, followed by incubation with primary and secondary antibodies. Tris-buffered saline and Tween 20 (TBST) was used to wash the membranes for 15 min, which was repeated three times. Then, the bands were visualised using an enhanced chemiluminescence solution. Protein expression was observed using Molecular Imager Lab, and the densitometric analysis was performed by using ChemiDoc XRS (Bio-Rad, Hercules, CA, USA). Three independent experiments were performed independently for analysis.

### 4.12. Ethics Declaration

The research was performed in accordance with the Declaration of Helsinki, and the research program was reviewed by the ethics committee of the Chinese Academy of Medical Sciences (Beijing, China).

### 4.13. Statistical Analyses

All data are expressed as the mean ± standard error of the mean. When the data were normally distributed, they were analysed by unpaired two-tailed Student’s *t*-tests, multiple groups were analysed by one-way analysis of variance (ANOVA), and multigroup with two variables used two-way ANOVA. Data with equal variances were analysed by post hoc Bonferroni’s test, and data with unequal variance were analysed by Dunnett’s T3 test. When the data were not normally distributed, nonparametric tests were used. A *p* value < 0.05 was considered significant.

## Supplementary Materials

Supplementary materials can be found at http://www.mdpi.com/1422-0067/20/1/36/s1.
