# Supplementary material for: Protective Effects of Total Saponins of Aralia elata (Miq.) on Endothelial Cell Injury Induced by TNF-α via Modulation of the PI3K/Akt and NF-κB Signalling Pathways
Source: Int J Mol Sci. 2018 Dec 21;20(1):36. doi: 10.3390/ijms20010036 (PMC6337668; doi:10.3390/ijms20010036)
Supplement: Supplementary file 1 [file ijms-20-00036-s001.pdf]

Supplementary: Protective Effects of Total Saponins of *Aralia elata* (Miq.) on Endothelial Cell Injury Induced by TNF-α via Modulation of the PI3K/Akt and NF-κB Signalling Pathways

Ping Zhou [1,2,3,4], Weijie Xie [1,2,3,4], Yun Luo [1,2,3,4], Shan Lu [1,2,3,4], Ziru Dai [1,2,3,4], Ruiying Wang [1,2,3,4], Guibo Sun [1,2,3,4,*] and Xiaobo Sun [1,2,3,4]

1 Beijing Key Laboratory of Innovative Drug Discovery of Traditional Chinese Medicine (Natural Medicine) and Translational Medicine, Institute of Medicinal Plant Development, Peking Union Medical College and Chinese Academy of Medical Sciences, Beijing 100193, China; zhoup0520@163.com (P.Z.); xwjginseng@126.com (W.X.); xlZhang2022@163.com (Y.L.); ginseng123@163.com (S.L.); athenadai219@163.com (Z.D.); shengjupan@163.com (R.W.)
2 Key Laboratory of Bioactive Substances and Resource Utilization of Chinese Herbal Medicine, Ministry of Education, Beijing 100193, China
3 Key Laboratory of Efficacy Evaluation of Chinese Medicine against Glycolipid Metabolic Disorders, State Administration of Traditional Chinese Medicine, Beijing 100193, China
4 Zhongguancun Open Laboratory of the Research and Development of Natural Medicine and Health Products, Beijing 100193, China
* Correspondence: sunguibopaper@163.com (G.S.); sunxiaobopaper@163.com (X.S.); Tel.: +86-10-5783-3220 (G.S.); +86-10-5783-3013 (X.S.)

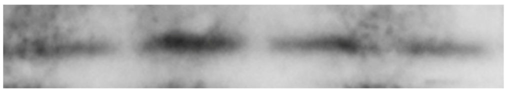

Figure S1. TAS treatment weakened the upregulation of caspase-8 induced by TNF-α treatment.

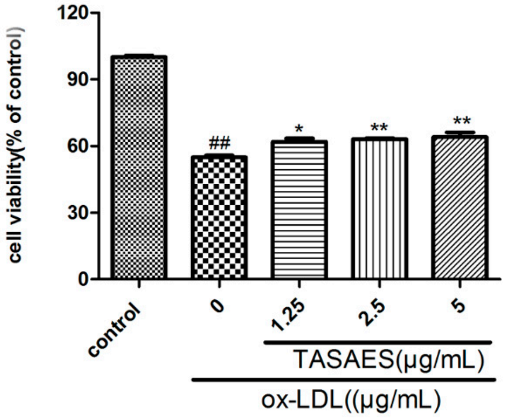

Figure S2. Protective effect against ox-LDL stimulus-induced endothelial cell injury.
